# Supplementary figures and images for: Small molecule inhibition of IRE1α kinase/RNase has anti-fibrotic effects in the lung
Source: PLoS One. 2019 Jan 9;14(1):e0209824. doi: 10.1371/journal.pone.0209824 (PMC6326459; doi:10.1371/journal.pone.0209824)

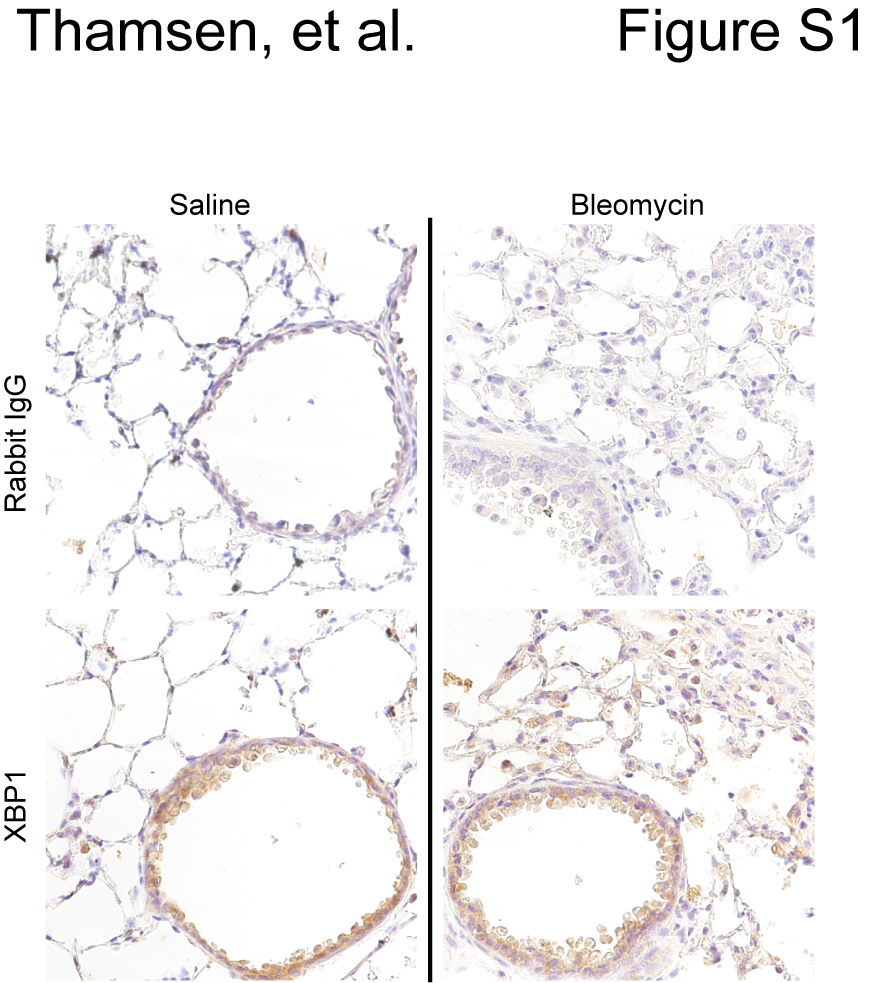

Supplement: S1 Fig — Immunohistochemical staining in saline- and bleomycin-exposed mouse lungs, using rabbit IgG control and anti-XBP1. (TIF) [file pone.0209824.s001.tif]

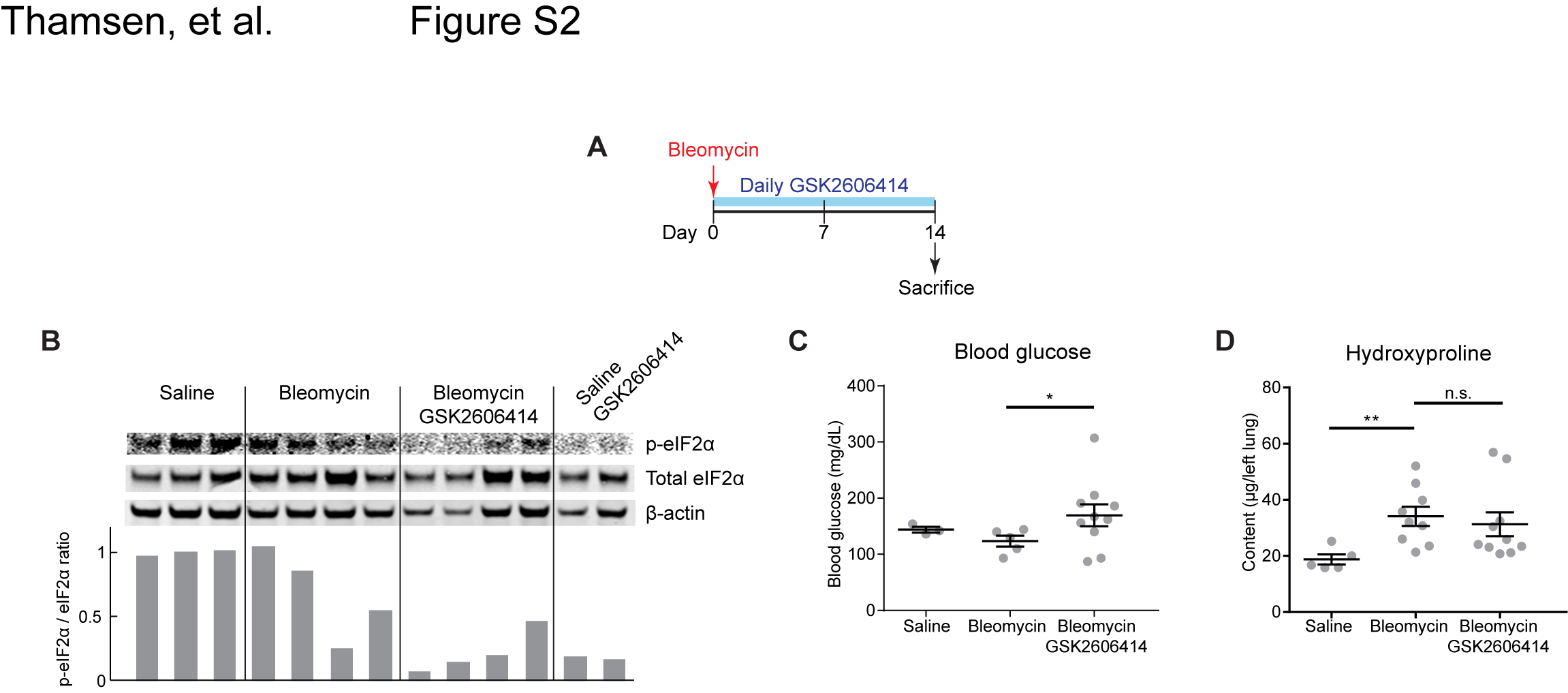

Supplement: S2 Fig — (A) Schematic of the GSK2606414 prevention regimen. Mice were exposed to saline or bleomycin once, then treated with GSK2606414 or vehicle beginning from the time of bleomycin exposure and daily for two weeks after exposure. (B) Western blot for phospho-eIF2α and total eIF2α from mice treated with GSK2606414 according to the reversal regimen (top) and quantification of phospho-eIF2α / total eIF2α ratio (bottom), normalized to saline-exposed lanes. (C) Blood glucose levels and (D) total hydroxyproline quantification from mice exposed to saline or bleomycin, and treated with GSK2606414 according to the reversal regimen. Each mouse is represented by a dot, and whiskers denote group mean +/- SEM. P values: *<0.05, **<0.01. (TIF) [file pone.0209824.s002.tif]
